# A novel coordination complex of platinum (PT) induces cell death in colorectal cancer by altering redox balance and modulating MAPK pathway

**DOI:** 10.1186/s12885-020-07165-w

**Published:** 2020-07-23

**Authors:** Khayal Al-Khayal, Mansoor-Ali Vaali-Mohammed, Mohammed Elwatidy, Thamer Bin Traiki, Omar Al-Obeed, Mohammad Azam, Zahid Khan, Maha Abdulla, Rehan Ahmad

**Affiliations:** 1 Colorectal Research Chair, Department of Surgery, King Saud University College of Medicine, PO Box 7805 (37), Riyadh, 11472 Saudi Arabia; 2 College of Medicine Research Center, King Saud University College of Medicine, Riyadh, 11472 Saudi Arabia; 3 Department of Chemistry, College of Science, King Saud University, Riyadh, 11451 Saudi Arabia; 4 Genome Research Chair, Department of Biochemistry, College of Science, King Saud University, Riyadh, Saudi Arabia

**Keywords:** Apoptosis, Platinum, Redox balance, Colorectal Cancer, MAPK

## Abstract

**Background:**

Colorectal cancer (CRC) is a heterogeneous tumor having various genetic alterations. The current treatment options had limited impact on disease free survival due to therapeutic resistance. Novel anticancer agents are needed to treat CRC specifically metastatic colorectal cancer. A novel coordination complex of platinum, (salicylaldiminato)Pt(II) complex with dimethylpropylene linkage (PT) exhibited potential anti-cancer activity. In this study, we explored the molecular mechanism of PT-induced cell death in colorectal cancer.

**Methods:**

Colony formation was evaluated using the clonogenic assay. Apoptosis, cell cycle analysis, reactive oxygen species, mitochondrial membrane potential and caspase-3/− 7 were assessed by flow cytometry. Glutathione level was detected by colorimetric assay. PT-induced alteration in pro-apoptotic/ anti-apoptotic proteins and other signaling pathways were investigated using western blotting. P38 downregulation was performed using siRNA.

**Results:**

In the present study, we explored the molecular mechanism of PT-mediated inhibition of cell proliferation in colorectal cancer cells. PT significantly inhibited the colony formation in human colorectal cancer cell lines (HT-29, SW480 and SW620) by inducing apoptosis and necrosis. This platinum complex was shown to significantly increase the reactive oxygen species (ROS) generation, depletion of glutathione and reduced mitochondrial membrane potential in colorectal cancer cells. Exposure to PT resulted in the downregulation of anti-apoptotic proteins (Bcl2, BclxL, XIAP) and alteration in Cyclins expression. Furthermore, PT increased cytochrome c release into cytosol and enhanced PARP cleavage leading to activation of intrinsic apoptotic pathway. Moreover, pre-treatment with ROS scavenger N-acetylcysteine (NAC) attenuated apoptosis suggesting that PT-induced apoptosis was driven by oxidative stress. Additionally, we show that PT-induced apoptosis was mediated by activating p38 MAPK and inhibiting AKT pathways. This was demonstrated by using chemical inhibitor and siRNA against p38 kinase which blocked the cytochrome c release and apoptosis in colorectal cancer cells.

**Conclusion:**

Collectively, our data demonstrates that the platinum complex (PT) exerts its anti-proliferative effect on CRC by ROS-mediated apoptosis and activating p38 MAPK pathway. Thus, our findings reveal a novel mechanism of action for PT on colorectal cancer cells and may have therapeutic implication.

## Background

Colorectal cancer is the 3rd most common malignancy globally and 2nd leading cause of cancer related death [1]. Globally 1.8 million new CRC cases are diagnosed annually [2]. The development of colorectal cancer involves three major routes, adenoma to carcinoma, inflammatory pathway and serrated pathway [1]. Most of colorectal cancers are adeno-carcinoma that arises from glandular intestinal epithelial cells of the colorectum [3]. Colorectal cancer constitutes an aetiologically heterogeneous disease categorized by tumor location and global gene alterations [4]. Approximately 60% of colorectal cancer cases develop sporadically without a family history of CRC along with increased CRC risk by inheriting genetic mutations [5]. The hereditary component of CRC involved around 35–40% cases [6]. Initially CRC was thought to be a disease of developed countries however, rapid increase in CRC incidence are occurring in developing countries undergoing economic development, diet and life style changes [2]. The increased incidence of CRC in younger population is an emerging trend [7].

The main therapeutic options for treating colorectal cancer are surgery, chemotherapy and radiotherapy [8–10]. Chemotherapy can be given to patients at different stages during CRC treatment. Mostly it is given after surgery as an adjuvant therapy with late stage disease. In some cases it is also given as neoadjuvant chemotherapy before surgery [11]. With the availability of various chemotherapeutics drugs, the overall survival of CRC patients has been improved for the past decades. However, drug resistance develops in nearly all patients with colorectal cancer and limits the drug efficacies of chemotherapeutic agents that lead to unsuccessful chemotherapy [12]. 5-Fluorouracil-based chemotherapy remains the main option for CRC patients [13]. However, in recent years, other chemotherapeutic agents have been developed like oxaliplatin, irinotecan and capecitabine. Mostly, treatment for advanced CRC disease involves combination of 5-FU and leucovorin with oxaliplatin or irinotecan [14]. With the advent of monoclonal antibodies like Cetuximab and Bevacizumab, CRC treatment has made better strides. Despite the better outcome with various combination strategy involving chemotherapy and monoclonal antibodies, the 5 year survival for advanced CRC disease is only over 12% [15]. Most of cancer related deaths are due to chemotherapy failure because of drug resistance [12]. Cisplatin is designated as a golden chemotherapeutic agent in solid tumor treatment. However, in colorectal cancer, therapy is limited by adverse effects, resistance and decreased effectiveness [16].

Mitogen-activated protein kinases are known to play essential role in cell proliferation, apoptosis, differentiation etc. [17]. There are three major MAPK pathways consisting of Erk1/2, JNK and MAPK14 p38 kinase. Erk1 and 2 undergo activation in response to cytokines and growth factors [18]. JNK pathway is activated in response to radiation, growth factors and environmental stress and is involved in regulating stress, apoptosis and inflammation. P38 (MAPK14) play important role in autoimmunity and activated by stress and cytokines like IL1 and TNFα targeting NFkB and p53 transcription factors [19].

The enhancement of efficacy by specific compounds may provide a valuable contribution to the treatment of colorectal cancer based on cisplatin chemotherapy. Recently, coordination chemistry of a platinum complex based on salen ligand ((salicylaldiminato)Pt(II) complex with dimethylpropylene linkage) exhibited potential antiproliferative activity [20]. In this study, we explored the mechanism of action of this novel platinum complex (PT) in colorectal cancer cells. The data indicated that PT inhibited the colony formation and induced ROS-mediated apoptosis. Importantly, we demonstrated that PT-mediated apoptosis ensues through activation of p38 MAPK.

## Methods

### Cell culture

Human adenocarcinoma colorectal cancer cell lines HT-29 (HTB-38), SW480 (CCL-228) and metastatic colorectal cancer cell line SW620 (CCL-227) were purchased from ATCC (Manassa VA, USA). HT-29 and SW620 cells were maintained in RPMI-1640 media containing 10% heat-inactivated fetal bovine serum (Thermo Fisher Scientific Inc., Waltham, MA USA), 100 Unit/ml penicillin (Thermo Fisher Scientific Inc. Waltham, MA USA), and 2 mM L-glutamine (Thermo Fisher Scientific Inc., Waltham MA USA). SW480 cells were cultured in DMEM media having 10% heat-inactivated fetal bovine serum (Thermo Fisher Scientific Inc., Waltham, MA USA), 100 Unit/ml penicillin (Thermo Fisher Scientific Inc. Waltham, MA USA), and 2 mM L-glutamine (Thermo Fisher Scientific Inc., Waltham MA USA). New batches of cells were confirmed by the STR analysis. All the cell lines underwent mycoplasma testing. In certain experiments, N-Acetylcysteine (NAC) 5 mM, JNK inhibitor: SP600125 10 μM, p38 kinase inhibitor: SB202190 10 μM, PI3K inhibitor: LY294002 10 μM and MAP kinase inhibitor: U0126 10 μM were used (Santa Cruz Biotechnology Inc., Dallas TX USA).

### Clonogenic assay

Colony formation assay was done as previously described by Gamage et al. [21]. HT-29 and SW620 cells were harvested and re-suspended in RPMI 1640 media and DMEM media was used for SW480. The respective cells were seeded into 6-well plates at 500 cells/well containing 2.0 ml media and incubated for 4–6 h to allow attachment. Different concentration of platinum complex was added for 24 h. Next day media containing PT was replaced with fresh medium and further incubated for 10–12 days in CO_2_ incubator. Colonies were fixed using 4% paraformaldehyde and staining was carried out by 0.05% crystal violet. The colonies were quantified under a microscope.

### Flow Cytometry analysis for apoptosis and cell cycle

Drug induced cell death consisting of apoptosis and necrosis was determined as reported by Ahmad et al. [22]. Briefly, Human colorectal cancer cell lines were seeded into 6-well plate at a density of 1 X 10^5^ /well and cultured for 24 h. Next day these cells were treated with different concentration of platinum complex for 24 h. Then the cells were harvested and washed twice with cold PBS. Detection of cell death was carried out by Annexin V/ Dead cell apoptosis kit (Cat# V13242, Thermo Fisher Scientific Inc., Waltham MA USA). The cells were resuspended in 1X binding buffer and incubated with Annexin V-FITC (5 μl) and 1 μl propidium iodide for 15 min in dark at room temperature. The acquisition and analysis of data were performed using CellQuest Pro Ver 6.0 BD FACSCALIBUR (BD Biosciences, San Jose CA USA). For cell cycle analysis, PT-treated cells were fixed in 70% ethanol, washed and incubated with RNase (200 μg/ml) (Thermo Fisher Scientific Inc., Waltham MA USA) for 30 min at room temperature. Samples were stained with propidium iodide (PI 50 μg/ml Thermo Fisher Scientific Inc., Waltham MA USA). Measurements of DNA content were made by flow cytometry (BD Biosciences, San Jose CA USA).

### Reactive oxygen species (ROS) measurement

Generation of ROS in response to drug treatment was done as mentioned earlier [22]. Colorectal cancer cell lines were cultured reaching 60% confluency. Next day they were treated with PT for 24 h. After incubation, cells were harvested and washed two times with cold phosphate buffered saline (PBS). 1.0 μM of 2′,7′-dicholorodihydrofluorescein diacetate (DCF-DA) was added to all samples and incubated for 15 min at 37 °C. After incubation all the samples were washed twice with PBS and analyzed for ROS detection by flow cytometry (BD FACSCALIBUR, BD Bioscences, San Jose CA, USA). Post-acquisition of data, analysis was done by CellQuest Pro Ver 6.0 using excitation at 488 nm and detection at 535 nm.

### Measurement of Total glutathione

Total glutathione levels were measured using a glutathione assay kit (Cayman Chemical Co. Ann Arbor, MI, USA). Cells were treated with 10 μM PT for 24 h at 37 °C. Cells were harvested and washed twice with PBS. The cell pellets were homogenized in 50 mM MES buffer containing 1 mM EDTA and centrifuged at 10,000×g for 15 min in the cold. The supernatants were mixed with assay cocktail along with standards in 96-well plates and incubated for 25 min. The absorbance was measured using the end-point method at 405 nm.

### Mitochondrial membrane potential analysis

All the cells were grown to 50–60% confluency. Next day they were treated with PT drug for 24 h at 37 °C. Then, cells were harvested, washed twice with PBS and incubated with rhodamine 123 (25 ng/ml) in PBS (Molecular Probes, Thermo Fisher Scientific, Waltham, MA USA) for 20 min at 37 °C. Rhodamine positive cells were detected using flow cytometry [23].

### Caspase-3/7 activity detection

The human colorectal cancer cell lines were treated with different concentrations of PT drug complex and incubated for 24 h at 37 °C. For detecting caspase-3 and -7 activities, we employed the Vybrant FAM Caspases assay kit (Thermo Fisher Scientific Inc., Waltham, MA USA) [22]. After drug treatment, cells were harvested and washed twice with PBS and incubated with FLICA for 60 min in CO_2_ incubator. Subsequently the cells were washed twice with washing buffer. Further the cells were incubated with propidium iodide (PI) for 5–10 min and analyzed using BD FACSCALIBUR (BD Biosciences, San Jose, CA USA).

### Western blotting

HT-29 and SW620 cells were cultured in RPMI medium and treated with PT for 24 h. Next day, the cells were harvested and washed twice with PBS. Total cell lysates were prepared using RIPA buffer (radioimmunoprecipitation assay lysis buffer (Boston Bioproducts, Ashland MA USA) by incubating cell pellets in cell lysis buffer for 15 min at 4 °C [22]. The whole mixture was centrifuged at 14000 rpm for 15 min. After centrifugation supernatant having the soluble proteins was collected and the concentration of proteins was measured on Bio-Rad SmartSpec Plus spectrophotometer using Bradford protein assay reagent (Bio-Rad Laboratories, Hercules, CA USA). 10–20 μg of protein were loaded on electrophoresis gels (4–20% Mini-Protean TGX precast gels) (Bio-Rad Laboratories, Hercules, CA USA). The separated proteins on precast gels were transferred to 0.2 μm PVDF membrane (Trans-Blot Turbo transfer pack, Bio-Rad Laboratories, Hercules, CA USA) using turbo protein transfer system (Bio-Rad Laboratories, Hercules, CA USA). Subsequently, the membranes with transferred proteins were blocked in Sea Block blocking buffer (Product# 37527 Thermo Scientific, Waltham MA USA) for 1 h at room temperature. Next, the membranes were washed twice with PBS containing 0.1% Tween-20 (PBST). Then the membranes were added with the following primary antibodies for Bcl2 (sc-492), BclxL (sc-56,021), XIAP (sc-58,537), Cyclin D1 (sc-8396), Cyclin E1 (sc-248), cytochrome c (sc-13,156), cleaved PARP1 (sc-56,196), P-p38 (sc-7973), p38 (sc-7972), P-AKT (sc-271,966), AKT (sc-5298), P-JNK (sc-6254), JNK (sc-7345), P-Erk (sc-7383), Erk (sc-135,900) and β-actin (sc-47,778) were purchased from Santa Cruz Biotechnology Dallas TX USA. P-Hsp27 (TA325546) and Hsp27 (TA890054) were purchased from Origene, Rockville MD USA. After overnight incubation with above antibodies on orbital shaker at 4 °C, the membranes were washed twice with PBST followed by incubation with HRP-conjugated mouse secondary and rabbit secondary antibodies (1:3000 dilution sc-516,102; sc-2357 Santa Cruz Biotechnology Dallas TX USA) on orbital shaker for 1 h at 25 °C. Detection of chemiluminescence signal was done by adding equal volume of detection reagent 1 and 2 (Thermo Fisher Scientific Waltham MA USA) and incubating for 5 min at room temperature. Signals were detected on C-DiGit blot scanner (LI-COR Biotechnology, Lincoln NE USA).

### Preparation of cytosolic extract

HT-29 and SW620 cells were treated with different concentration of PT for 24 h. Cytosolic extracts were prepared using kit (Cytochrome c releasing apoptosis assay kit Cat# ab65311). Harvested cells were washed twice with ice-cold PBS. Cells were re-suspended in 1X cytosolic extraction buffer containing DTT and protease inhibitors and incubated on ice for 10 min and homogenized by Dounce tissue grinder on ice. Homogenate was centrifuged at 700Xg for 10 min and then collected supernatant was further centrifuged at 10,000Xg for 30 min at 4 °C. Collected supernatant was considered as cytosolic extract and used for cytochrome c detection.

### Transient transfection for p38 siRNA

SW620 cells were grown to 50–60% confluency. Next day, Lipofectamine RNAi/Max (Thermo Fisher Scientific, Waltham MA USA) and control siRNA duplex (Cat#30004 Origene, Rockville MD USA); p38siRNA duplex (SR301010 Origene, Rockville MD USA) were diluted in Opti-MEM medium (Thermo Fisher Scientific, Waltham MA USA). Diluted siRNAs and Lipofectamine RNAi/Max reagent were mixed together (1:1 ratio) and incubated for 5 min. Complete media was removed and siRNA-lipid complex was added to cells. Cells were incubated for 48–72 h at 37 °C before analyzing p38 protein expression.

### Statistical analysis

Results are presented as mean of three independent experiments (mean ± SD). GraphPad Prism7 (GraphPad Software Inc) was used for statistical analysis. Differences between the control and treated group were compared using One-way ANOVA statistical test. *P* values < 0.05 were considered statistically significant.

## Results

### PT inhibits colony formation

**Fig. 1:**
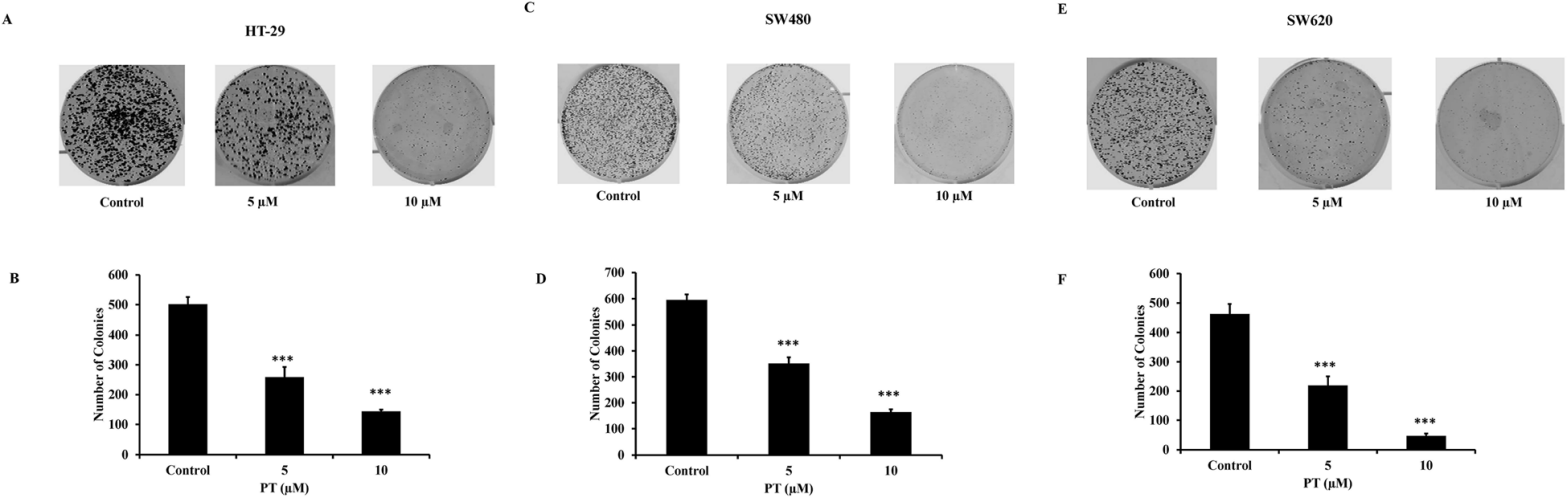
PT inhibits colony formation. **a-b** HT-29 **c-d** SW480 **e-f** SW620 cells were seeded as single cell at 500 cells/well in 6-well plate. After 4–6 h, PT (5 and 10 μM) was added for 24 h and incubated at 37 °C. After 24 h media containing PT was replaced with fresh complete media and cells were further incubated for 10–12 days for colony at 37 °C. Crystal violet staining was done and colonies were quantified using light microscope and images were captured by Bio-Rad Gel-Doc system. Results are shown as representative of three independent experiment (*n* = 3). ***p < 0.001 PT (5) vs control; ***p < 0.001 PT (10) vs control

To study the anticancer potential of PT, we employed two adenocarcinoma colorectal cancer cell line namely HT-29 and SW480; and a metastatic colorectal cancer cell line SW620. SW480 was derived from primary adenocarcinoma while SW620 was derived from a lymph node metastasis from the same patient giving rise to adenocarcinoma stage and metastatic stage respectively. Our previous finding [20] reported that PT was found to have IC50 of 5 μM and 7.5 μM for HT-29 and SW620 cells respectively. To explore the anticancer activity of platinum complex (PT), we tested the effect of PT at 5 and 10 μM on in-vitro tumorigenicity of HT-29, SW480 and SW620 cells. Treatment of HT-29 cells with different concentration of PT resulted in the inhibition of number of colonies, confirming our previous report [20] that the proliferation of the cells was depleted at these concentrations (Fig. 1a-b). Similar result was obtained in other adenocarcinoma cell line SW480 (Fig. 1c-d). The response of major anticancer drug for metastatic colorectal cancer patients is poor. To see the effect of this platinum complex on metastatic cells, we tested its efficacy on human metastatic colorectal cancer cell line SW620. PT treatment of SW620 cells resulted in the reduction of colony formation (Fig. 1e-f). These results demonstrate that this platinum complex has anti-tumorigenic activity in human colorectal cancer cell lines.

### PT induces apoptosis and cell cycle arrest in colorectal cancer cells

**Fig. 2:**
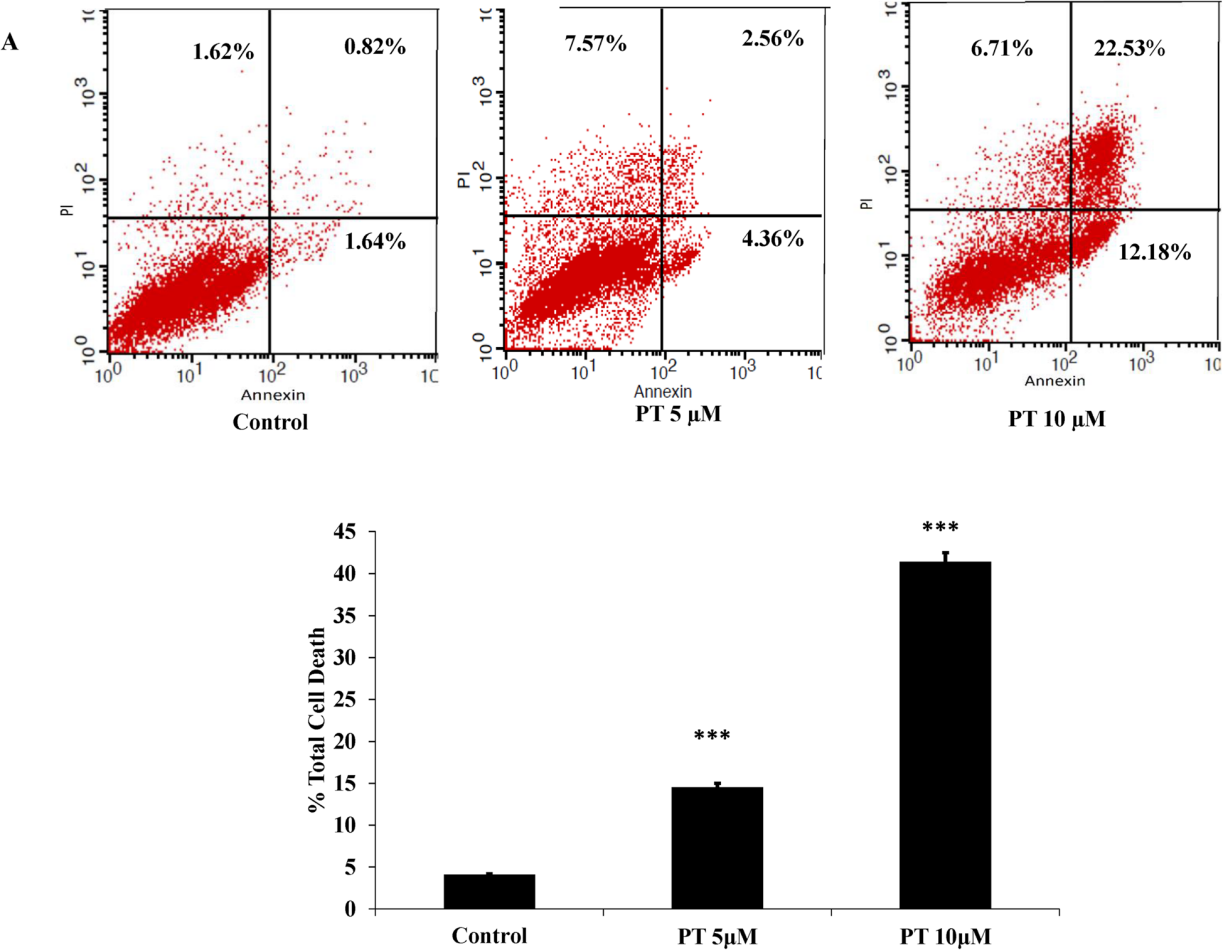
PT induces apoptosis and cell cycle arrest. **a** HT-29 **b** SW480 **c** SW620 cells were treated with 5 and 10 μM of PT for 24 h. Total cell death including apoptosis and necrosis was analyzed by Annexin V/PI staining using flow cytometry. **d** HT-29 and **e** SW620 cell cycle distribution was measured by PI staining using flow cytometry and the percentage of cell population was determined in the G0/G1, S and G2/M phases. Results shown are representative of three independent experiment (*n* = 3). *p < 0.05, **p < 0.01, ***p < 0.001 vs control

PT has been shown to inhibit cell viability of human colorectal cancer cell lines [20]. To determine whether PT-mediated cell death was induced by apoptosis, the effect of PT on the induction of apoptosis was analyzed by flow cytometry in different human colorectal cancer cell lines. Treatment of HT-29 with different concentration of PT was associated with increased cell death. As shown in Fig. 2a-b, PT was found to induce 14.5 and 41.4% apoptosis at 5 and 10 μM concentrations respectively as compared to control cells (4.08%). The effect of PT-induced cell death was analyzed in SW480. PT treatment of SW480 cells was associated with 32.7 and 68.8% total cell death as compared to 1.07% in the control cells at 5 and 10 μM concentrations (Fig. 2c-d). Furthermore, effect of PT-mediated cell death was studied in metastatic colorectal cancer cell lines SW620. As shown in Fig. 2e-f, incubation with 5 μM and 10 μM of PT for 24 h induced 24.7 and 41.7% total cell death in SW620 cells. To investigate, whether cell cycle arrest contributed to cell growth suppression by PT, we treated HT-29 cells with different concentration of PT for 24 h. Cell cycle distribution was measured by PI staining and the DNA content was examined by flow cytometry. PT was found to induce significant increase in cell population at the G2/M phase along with decrease in G0/G1 phase in a dose dependent manner (Fig. 2d). Similar result was obtained in SW620 cells (Fig. 2e). Collectively, PT was found to induce apoptosis and cell cycle arrest in dose dependent manner in human colorectal cancer cell lines.

### PT alters the redox balance

**Fig. 3:**
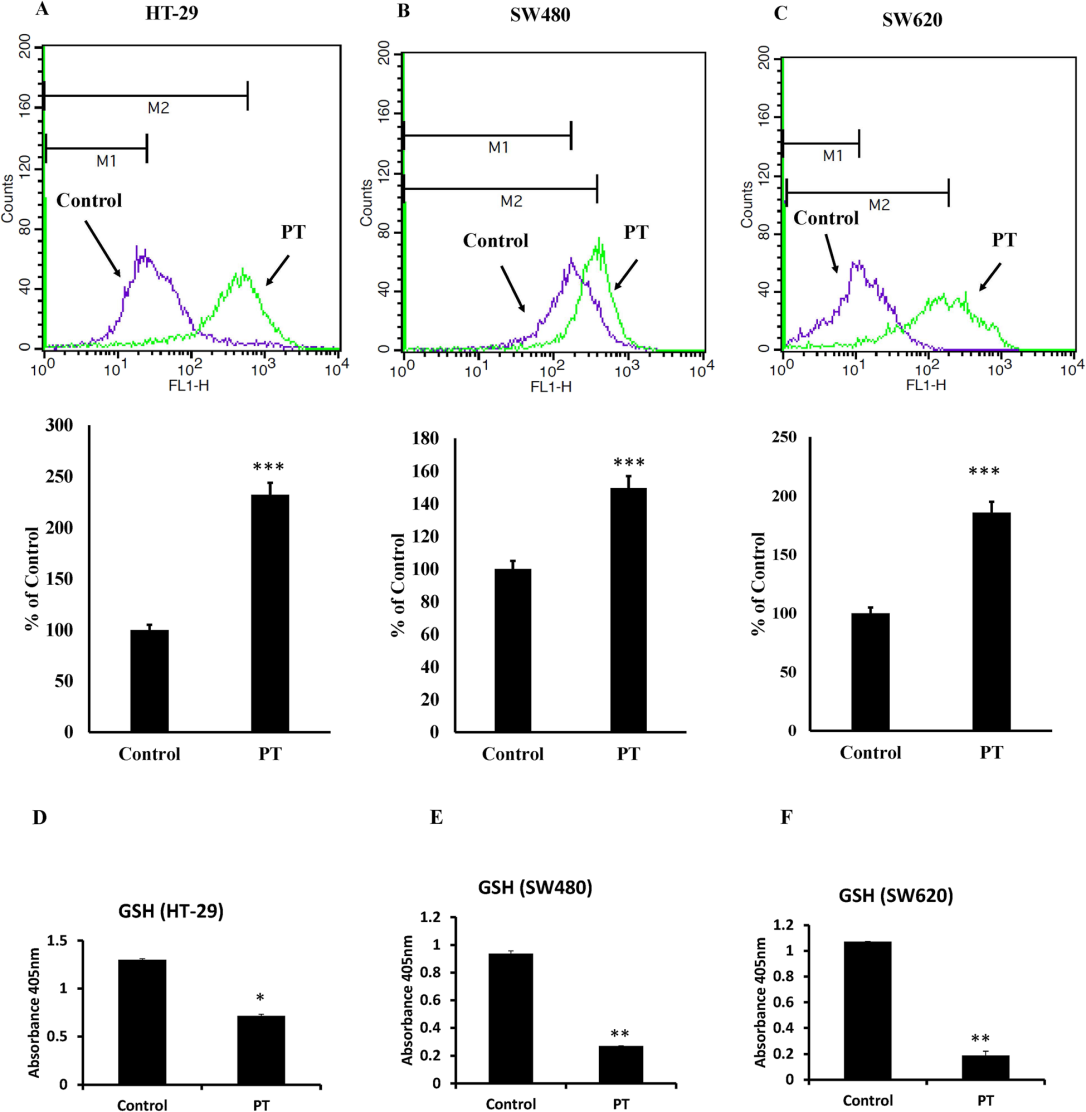
PT alters redox balance. **a** HT-29 **b** SW480 **c** SW620 cells were exposed to PT for 24 h. Cells were incubated with 2′,7′–dichlorofluorescein diacetate (DCFDA) for 15 min and the fluorescence of the oxidized 2′,7′–dichlorofluorescein was detected by flow cytometry. **c** HT-29 **d** SW480 **e** SW620 were treated with PT for 24 h. The absorbance was detected at 405 nm using plate reader. The bar graphs are presented as mean ± SD of three independent experiments. *p < 0.05, **p < 0.01, ***p < 0.001 vs control

It has been long recognized that oxidative stress is a common feature of apoptosis [24]. ROS production by aerobic cells can affect various cellular processes thereby leading to cell death under stress [25]. Most of the anticancer agents are known to induce reactive oxygen species production which results in DNA damage leading to cell death [25]. Incubation of PT with human colorectal cancer cell line HT-29 resulted in the increased production of ROS (Fig. 3a). Similar result was obtained in another adenocarcinoma cell line SW480 (Fig. 3b). We sought to know whether PT also induces ROS production in metastatic colorectal cancer cell line SW620. Indeed treatment of SW620 cells with PT resulted in enhanced generation of ROS (Fig. 3c). Reduced glutathione (GSH) is an essential thiol in protecting cells against toxic reactive oxygen species [26]. Physiologically GSH plays important role in controlling gene expression related to apoptosis, membrane transport and drug resistance to chemotherapy [27, 28]. Treatment of human colorectal cancer cell lines with PT resulted in the depletion of total glutathione levels (Fig. 3d-f). These findings demonstrate that this platinum complex alters redox balance by generation of ROS and depletion of glutathione in colorectal cancer cells.

### Mitochondrial membrane potential modulation by PT

**Fig. 4:**
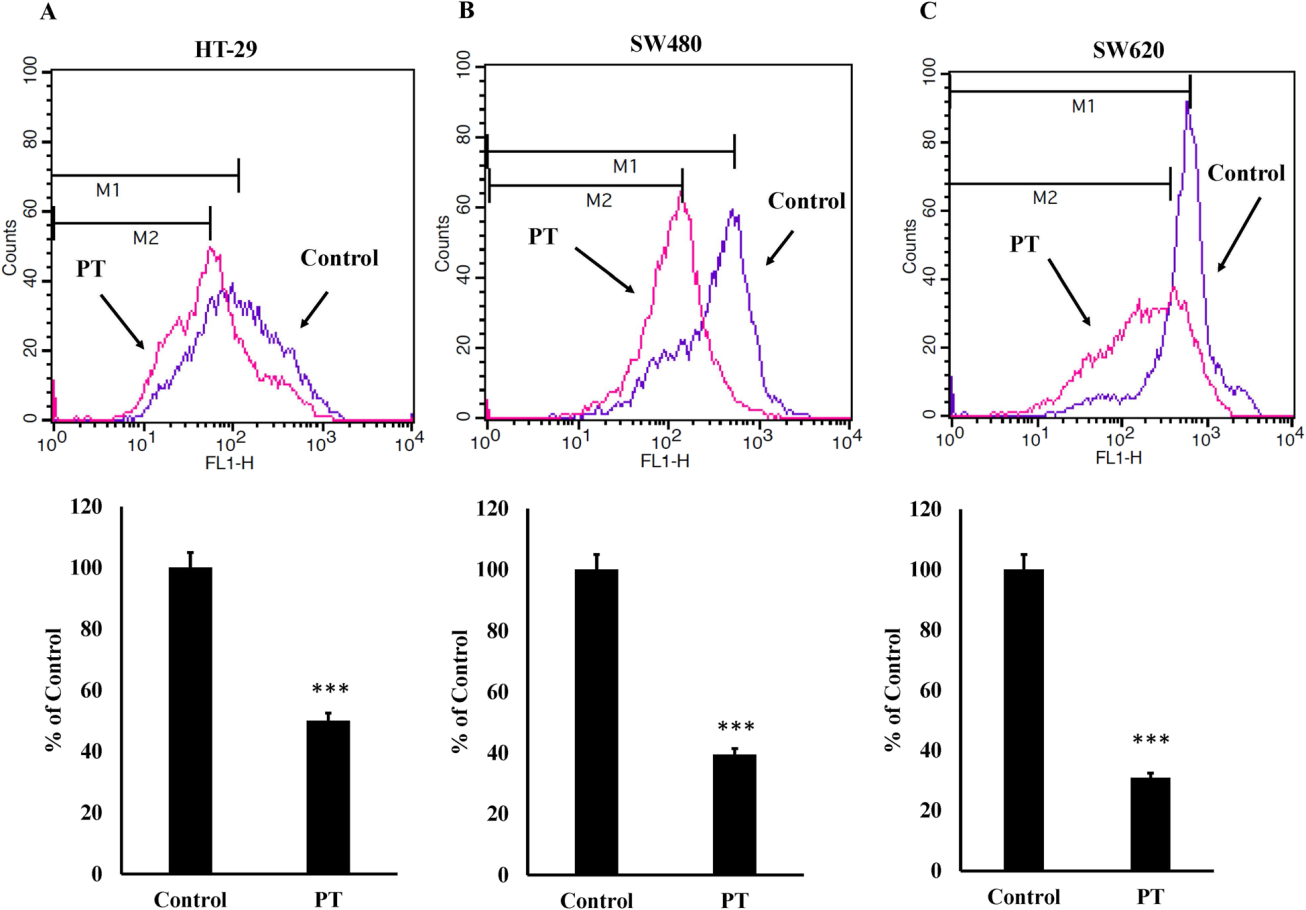
PT inhibits mitochondrial membrane potential. **a** HT-29 **b** SW480 **c** SW620 cells were treated PT for 24 h. Cells were washed with PBS and incubated with rhodamine 123 (25 ng/ml) at 37 °C for 20 min. Positive stained cells for rhodamine 123 were analyzed by flow cytometry. The bar graphs are presented as mean ± SD of three independent experiments. ***p < 0.001 vs control

Decrease in mitochondrial membrane potential leads to the release of cytochrome c into cytosol culminating into apoptosis [29]. To investigate whether PT treatment results in the alteration in mitochondrial membrane potential, incubation of PT with HT-29 cells resulted in the decreased mitochondrial membrane potential (Fig. 4a). Similar results were obtained in other adenocarcinoma colorectal cancer cells SW480 (Fig. 4b) and metastatic colorectal cancer cells SW620 (Fig. 4c). This finding indicates that PT exerts its anticancer effect by decreasing mitochondrial membrane potential.

### PT inhibits anti-apoptotic gene expression

**Fig. 5:**
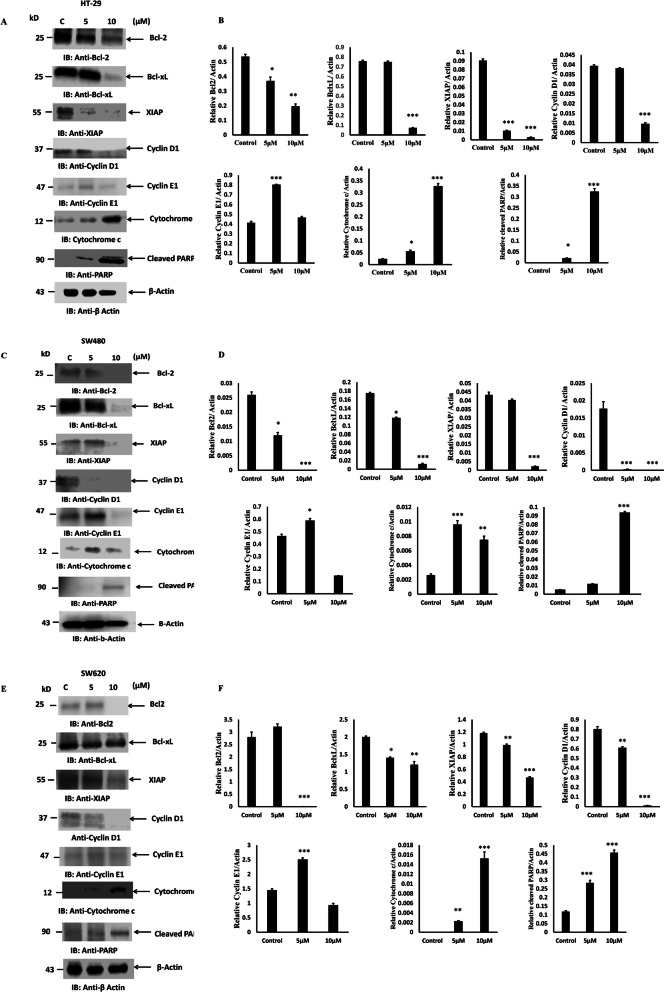
PT blocks anti-apoptotic protein expression and activates cytochrome c and PARP cleavage. **a** HT-29 **c** SW480 **e** SW620 cells were exposed to different concentration of PT for 24 h at 37 °C. Soluble fraction of total cell lysates were immunoblotted with indicated antibodies. Cytosolic fraction of HT-29, SW480 and SW620 were prepared and immunoblotted for cytochrome c. **b**, **d**, **f** Density of the protein bands of three independent experiments were quantified and expressed as relative protein expression to actin. The bar graphs are presented as mean ± SD of three independent experiments. *p < 0.05, **p < 0.01, ***p < 0.001 vs control. Full Length blots were presented in Supplementary Figure S1

To evaluate the effect of PT on cellular apoptosis, we determined the pro-apoptotic and anti-apoptotic protein expressions after PT treatment. Western blotting revealed that incubation with PT at 5 and 10 μM for 24 h was associated with downregulation of Bcl2 and BclxL in HT-29 cells (Fig. 5a-b). X-Linked inhibitor of apoptosis protein (XIAP) has been known to block apoptosis [30]. Treatment of HT-29 with different concentration of PT was associated with depletion of XIAP protein levels (Fig. 5a-b). Similarly during stress cyclins are known to regulate cell cycle leading to cell cycle arrest [31]. To evaluate the effect of PT on Cyclin D1 and Cyclin E1, HT-29 cells were treated with different concentration of PT for 24 h. As shown in Fig. 5a-b, PT was found to inhibit the expression of Cyclin D1 and increase the expression of Cyclin E1 at 5 μM. Alteration in mitochondrial membrane potential results in the release of cytochrome c [32]. PT-treated cytosolic extract was found to contain higher amount of cytochrome c as compared to control (Fig. 5a-b). Poly (ADP-ribose) polymerase (PARP) belongs to a family of proteins known to regulate cellular processes like DNA repair, genomic stability and programmed cell death [32]. PARP also act as a substrate for caspase-3 which cleaves to smaller fragment. PT-treatment of human colorectal cancer cells was associated with increased activation of PARP (Fig. 5a-b). Similar results were obtained in PT-treated SW480 (Fig. 5c-d) and SW620 cells (Fig. 5e-f).

### PT activates intrinsic apoptotic pathway

**Fig. 6:**
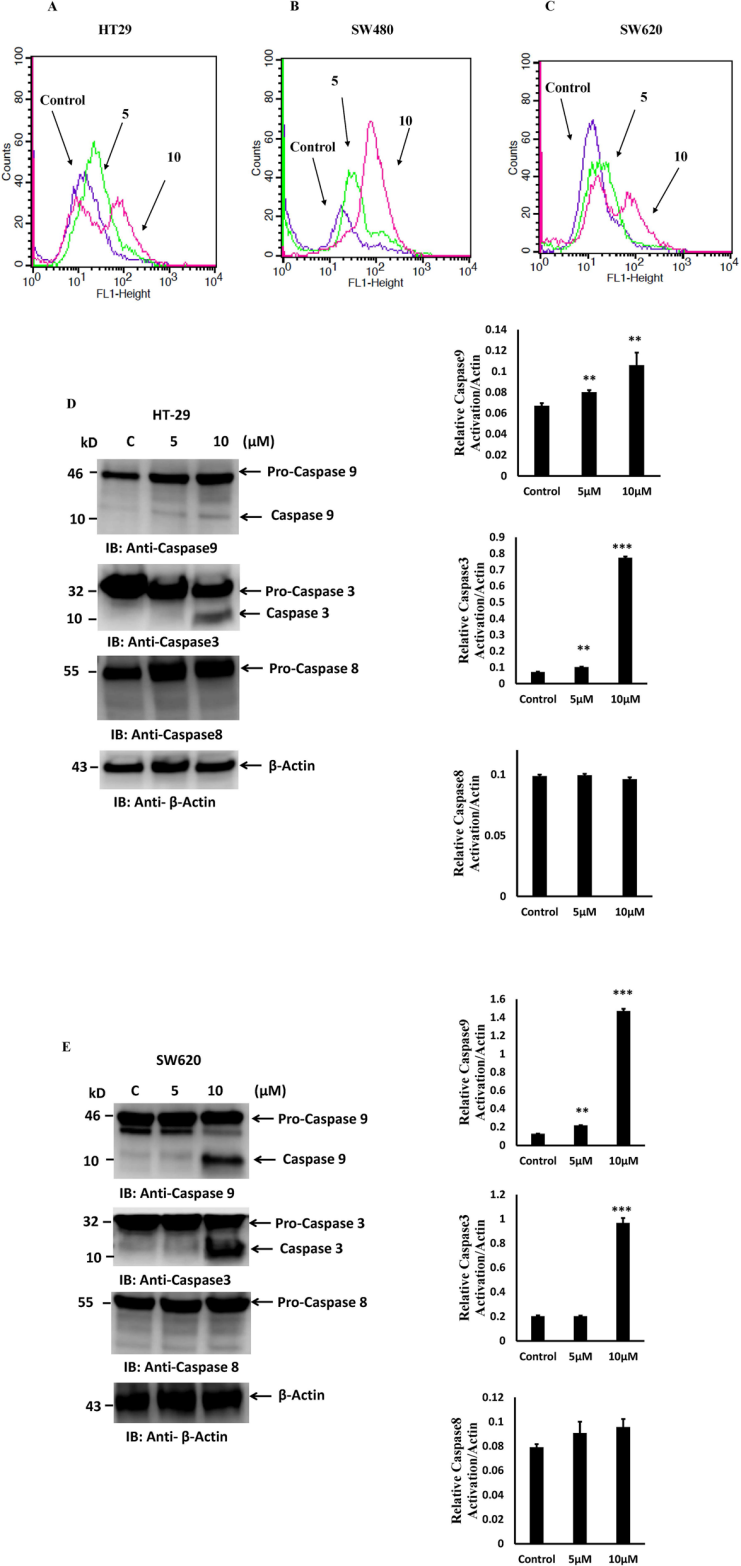
PT activates intrinsic apoptotic pathway. **a** HT-29 **b** SW480 **c** SW620 cells treated with different concentration of PT for 24 h at 37 °C. Activation of caspase-3 and -7 was analyzed by flow cytometry. **d** HT-29 **e** SW620 cells were exposed to different concentration of PT for 24 h at 37 °C. Total cell lysates were immunoblotted with the indicated antibodies. Density of the protein bands of three independent experiments were quantified and expressed as relative protein expression to actin. The bar graphs are presented as mean ± SD of three independent experiments. **p < 0.01, ***p < 0.001 vs control. Full Length blots were presented in Supplementary Figure S2

Release of cytochrome c into cytosol forms a complex with Apaf1 and procaspase-9 called apoptosome resulting in the autoactivation of caspase-9 and activating downstream caspase cascade [33]. HT-29 cells were treated with different concentration of PT for 24 h. Harvested cells were measured for caspase-3 and caspase-7 activity using flow cytometry. As indicated, PT treatment resulted in the increased activity of caspase-3 and caspase-7 in dose dependent manner (Fig. 6a). Treatment of another colorectal cancer cell line SW480 with PT resulted in similar finding (Fig. 6b). Incubation of SW620 with different concentration of PT was associated with increased caspase-3 and -7 activities in dose dependent manner (Fig. 6c). These findings were confirmed by analyzing caspase-9 and caspase-3 cleavage using western blotting (Fig. 6d-e). To see whether extrinsic apoptosis was also involved in PT-mediated apoptosis. PT-treated HT-29 and SW620 cell lysates were immunoblotted for caspase 8 cleavage. PT was not found to activate caspase-8 cleavage confirming that PT-induced cell death only involved intrinsic apoptosis pathway (Fig. 6d-e).

### PT induces ROS-mediated apoptosis and modulates MAPK pathway

**Fig. 7:**
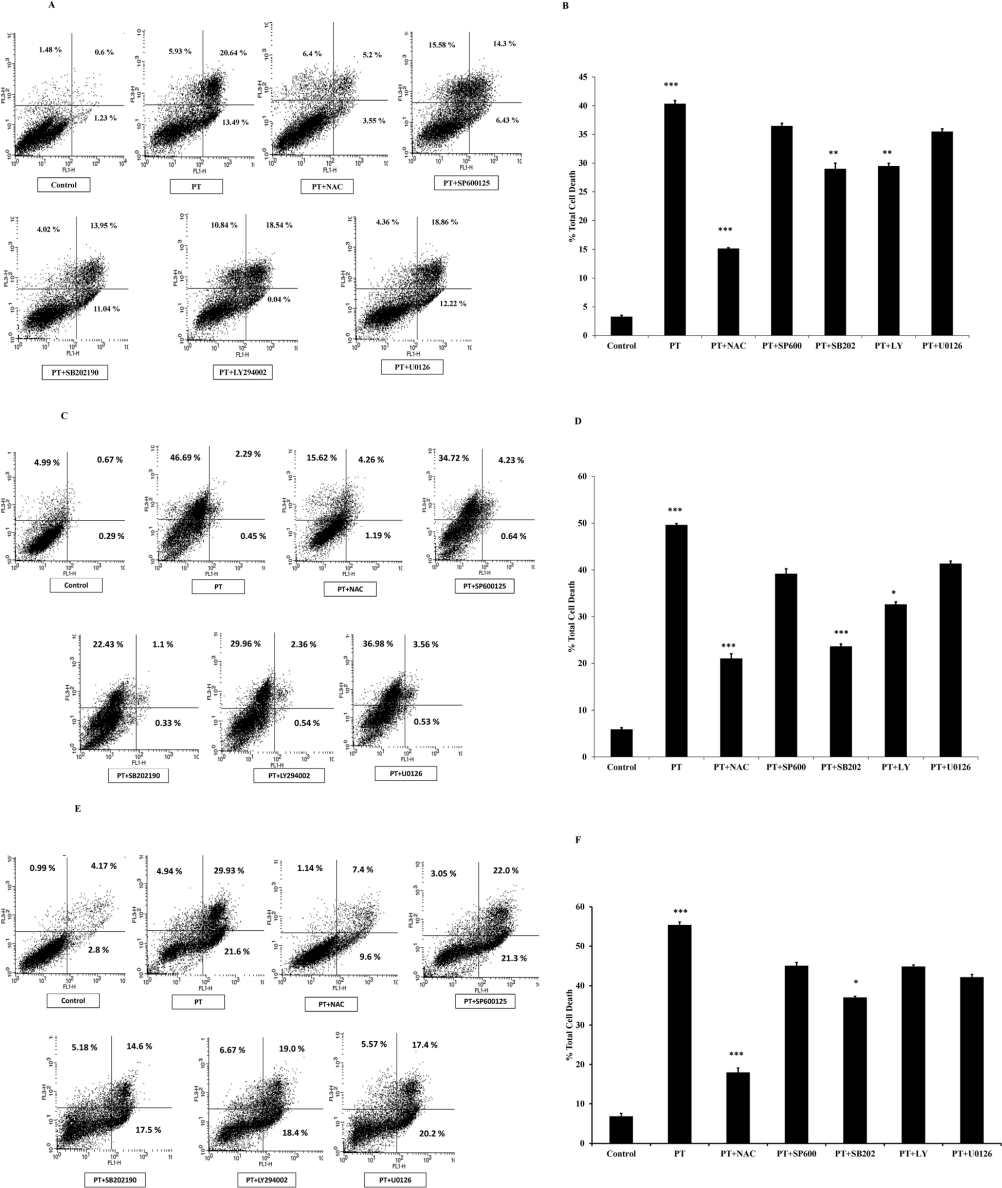
PT-induced apoptosis depends on ROS and p38 MAPK. **a** HT-29 **c** SW480 **e** SW620 cells were pre-treated with NAC (5 mM), SP600125 (10 μM), SB202190 (10 μM), LY294002 (10 μM) and U0126 (10 μM) for 1 h and then exposed to PT (10 μM) for 24 h at 37 °C. Total cell death was determined by Annexin V/PI staining using flow cytometry. The bar graph shows the percentage of total cell death and apoptotic/ necrotic cells and the results are presented as mean ± SD of three independent experiments. **b** %Total cell death ***p < 0.001 PT vs control and ****p < 0.001 PT + NAC vs PT signify a statistically significant difference. **p < 0.01 PT + SB202 vs PT; **p < 0.01 PT + LY vs PT statistically significant. **d** %Total cell death ***p < 0.001 PT vs control; ***p < 0.001 PT + NAC vs PT; ***p < 0.001 PT + SB202 vs PT; *p < 0.05 PT + LY vs PT. **f** % Total cell death ***p < 0.001 PT vs control; ***p < 0.001 PT + NAC vs PT; *p < 0.05 PT + SB202 vs PT

A number of anticancer agents activate MAPK signaling and ROS-mediated apoptosis in cancer cells [22, 34, 35]. To explore the molecular mechanism for the effect of platinum complex we utilized several kinase inhibitors. HT-29, SW480 and SW620 cells were pre-treated with ROS inhibitor: N-acetylcysteine (NAC), JNK inhibitor: SP600125, p38 kinase inhibitor: SB202190, AKT inhibitor LY294002 and ERK kinase inhibitor U0126 for 1 h followed by treatment with PT. We evaluated the total cell death induced by PT in response to various inhibitors. As shown in Fig. 7a-b, PT-induced significant cell death after 24 h treatment, however ROS scavenger (NAC) was found to alleviate total cell death induced by PT indicating that ROS generation was a critical step in PT-mediated cell death. Pre-incubation with p38 kinase inhibitor SB202190 was found to block significantly PT-mediated cell death in HT-29 cells. AKT inhibitor was also significantly found to block PT-induced cell death (Fig. 7a-b). Other kinase inhibitors had no significant effect on PT-mediated apoptosis. Similar experimentation was setup for other adenocarcinoma cell lines SW480. PT was found to induce cell death by inducing significant cell death. NAC was found to block total cell death in SW480 cells (Fig. 7c-d). Notably, p38 kinase inhibitor SB202190 and AKT inhibitor LY294002 significantly inhibited the total cell death induced by PT. JNK and ERK inhibitors had no significant effect on cell death in SW480 cells (Fig. 7c-d). We further investigated the effect of these inhibitors on PT-induced cell death in metastatic colorectal cancer cells SW620. NAC and SB202192 were found to block total apoptosis in response to PT (Fig. 7e-f). There was no significant effect of SP600125, LY294002 and U0126 on PT-induced cell death in SW620 cells. Collectively these findings suggest that PT-induced cell death was mediated by ROS, AKT and p38 kinase.

### PT modulates MAPK pathway

**Fig. 8:**
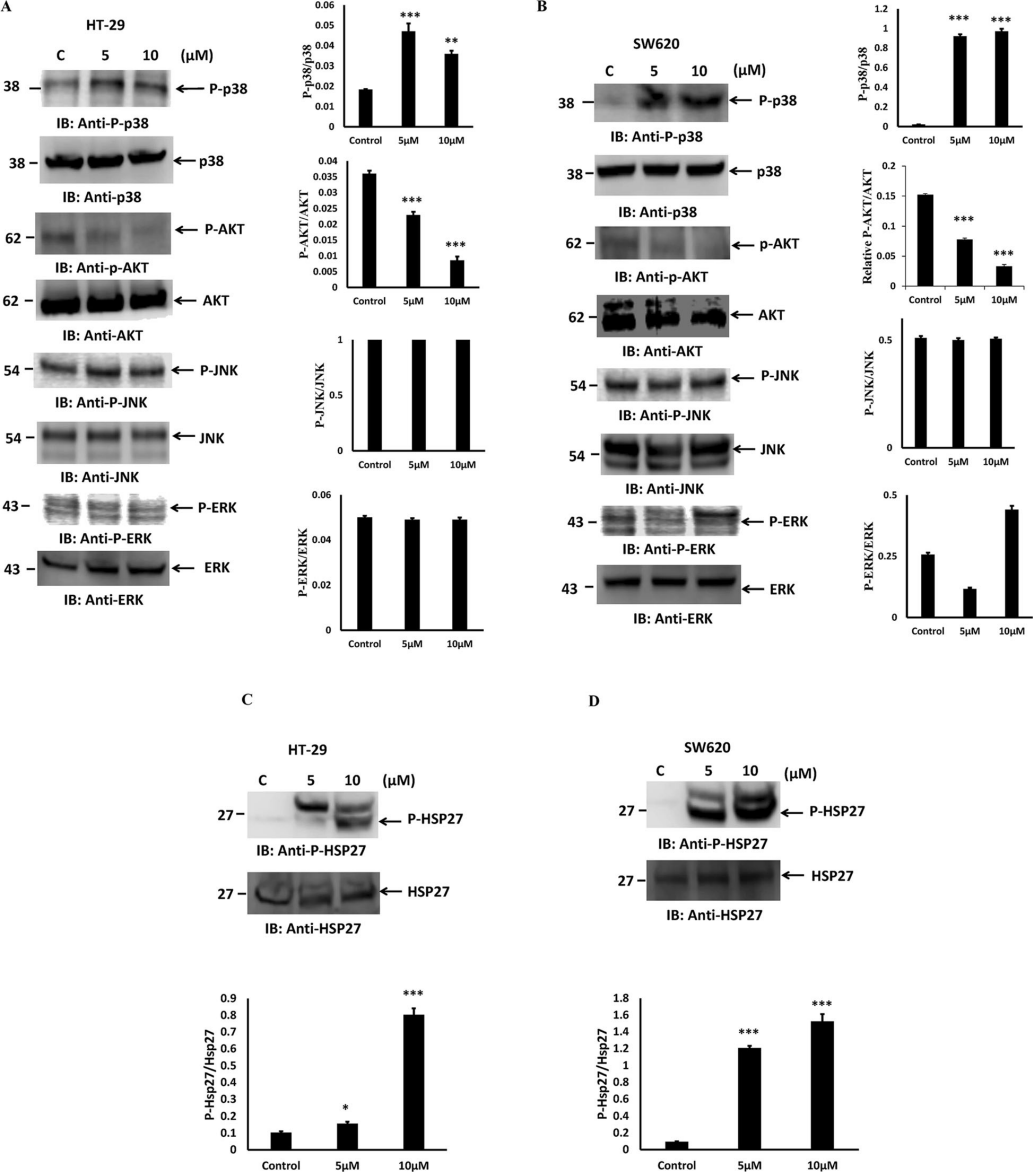
PT activates p38 MAPK and inhibits AKT phosphorylation. **a-c** HT-29 **b-d** SW620 cells exposed to different concentration of PT for 24 h at 37 °C. Total cell lysates were prepared and immunoblotted with the indicated antibodies. Density of the protein bands of three independent experiments were quantified and expressed as relative protein expression to actin. The bar graphs are presented as mean ± SD of three independent experiments. * < *p* < 0.05, **p < 0.01, ***p < 0.001 vs control. Full Length blots were presented in Supplementary Figure S3

P38 MAPK plays critical role in the regulation of cellular apoptosis [36, 37]. We evaluated the phosphorylation of different kinases of MAPK pathway after PT treatment. As shown in Fig. 8a, p38 MAPK phosphorylation was increased after PT treatment of HT-29 cells for 24 h. Phosphorylation of AKT was decreased in dose dependent manner in PT-treated HT-29 cells, whereas no significant alterations were observed in phosphorylation of JNK and ERK (Fig. 8a). Moreover, to further confirm this finding, SW620 cells were treated with PT for 24 h. Similar to HT-29, PT was found to increase p38 phosphorylation in SW620 cells. Phosphorylation of AKT was inhibited in response to PT treatment however no significant effect was noted on JNK phosphorylation. Though, PT was found to alter ERK phosphorylation in SW620 cells (Fig. 8b). To further elucidate the p38 kinase pathway we investigated the effect of PT on downstream target of p38. It is well recognized that Hsp27 is a downstream substrate of the p38 MAPK pathway [38, 39]. P38 MAPK is known to phosphorylate Hsp 27 on Serine-82 and alters its cellular distribution [40]. We evaluated the phosphorylation of Hsp27 in PT-treated HT29 cells. PT was found to increase the Hsp27 phosphorylation in a dose dependent manner in HT29 cells (Fig. 8c). Similar result was obtained in SW620 cell line (Fig. 8d). These findings thus confirmed that PT indeed activated p38 MAPK-Hsp27 pathway in colorectal cancer cells.

### PT-activates p38 kinase-mediated apoptosis

**Fig. 9:**
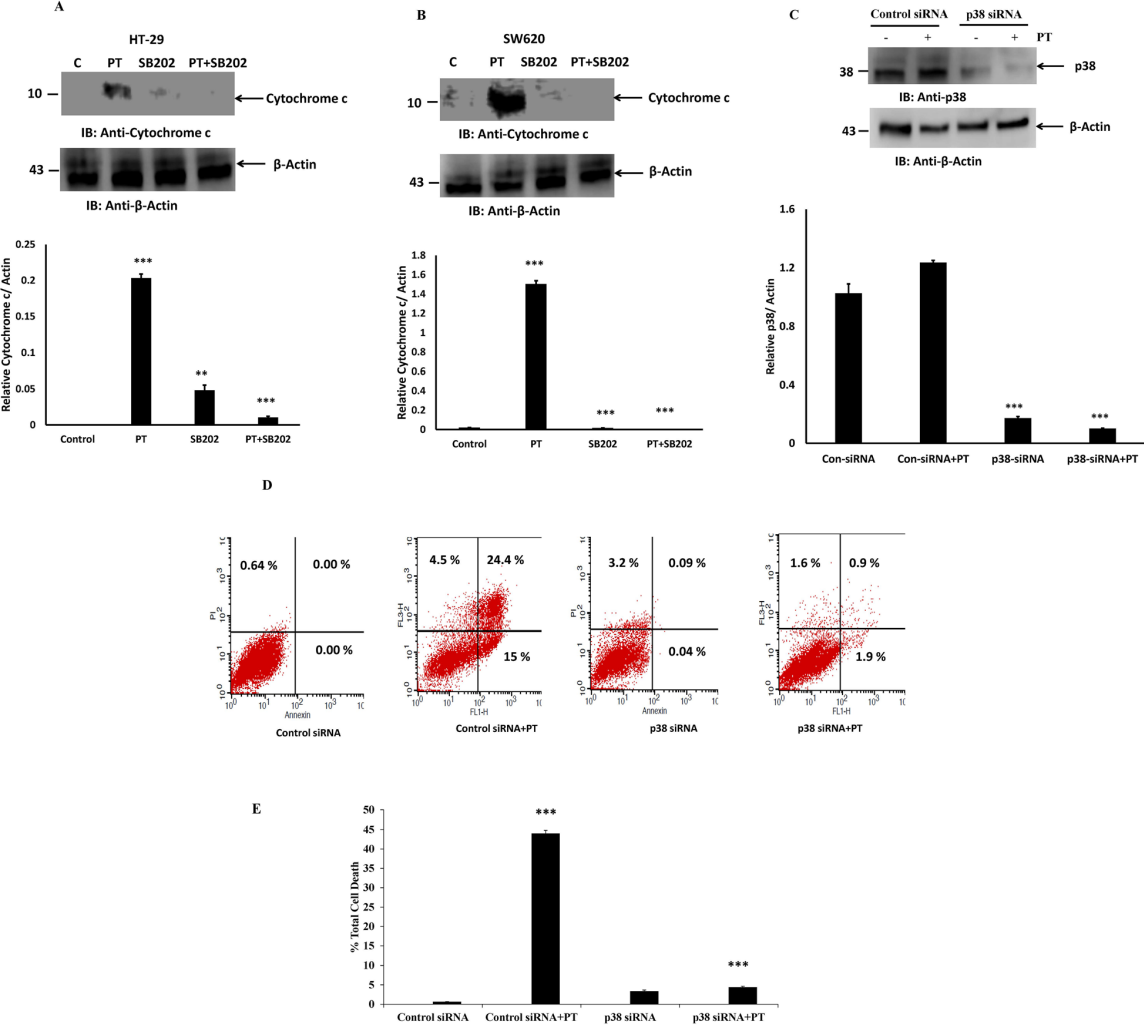
PT induces apoptosis by activating p38 MAPK. **a** HT-29 **b** SW620 cells were pre-treated with p38 MAPK inhibitor SB202190 (10 μM) for 1 h and then exposed to PT for 24 h at 37 °C. Cytosolic fraction were prepared and immunoblotted for the indicated antibodies. **c** SW620 cells were transfected with control and p38 siRNA duplex for 48 h then exposed to PT for 24 h at 37 °C. Total cell lysate were prepared and immunoblotted with indicated antibodies. Density of the protein bands of three independent experiments were quantified and expressed as relative protein expression to actin. The bar graphs are presented as mean ± SD of three independent experiments. **p < 0.01, ***p < 0.001 vs control. **d** SW620 cells were transfected with control and p38 siRNA duplex for 48 h then exposed to PT for 24 h at 37 °C. These cells were incubated with Annexin V/PI and %total cell death was detected by flow cytometry. **e** Results shown are representative of three independent experiments. ***p < 0.001 Control siRNA+PT vs Control siRNA; *** *p* < 0.001 p38siRNA + PT vs Control siRNA+PT. Full Length blots were presented in Supplementary Figure S4

We then examined whether p38 MAPK activation was necessary for PT-induced apoptosis in human colorectal cancer cells. HT-29 and SW620 cells were pre-treated with p38 kinase inhibitor SB202190 for 1 h followed by treatment with PT for 24 h. Cytosolic fraction were prepared and immunoblotted with cytochrome c. Notably, p38 kinase inhibitor SB202190 significantly inhibited PT-induced cytochrome c release into cytosol, indicating that SB202190 could alleviate PT-induced apoptosis in HT-29 (Fig. 9a) and SW620 cells (Fig. 9b). To confirm further the involvement of p38 kinase activation in PT-induced apoptosis, we used p38 siRNA to silence p38 protein expression in SW620 cells. As shown in Fig. 9c, p38 siRNA was found to inhibit the p38 protein expression in SW620 cells as compared to control siRNA. PT-induced apoptosis was significantly blocked in SW620 cells transfected with p38 siRNA as compared to PT-treated cells (Fig. 9d-e). These results thus indicate that PT-induced apoptosis was mediated by p38 MAPK pathway in colorectal cancer cells.

## Discussion

Cisplatin is a standard anticancer drug which has shown significant chemotherapeutic potential in treating various solid tumors [40]. However, cisplatin has shown side effects like renal failure, nausea, ear damage etc. [41, 42]. In most cancer chemotherapy, tumor cells develop drug resistance culminating into severe side effect leading to worst outcome [42, 43]. New advances in anticancer drug development by making alternative to standard known drugs for cancer therapeutics using coordination chemistry has been made in the last years [44, 45]. We reported earlier the development of platinum complex based on salen ligand (PT) having anticancer potential [20]. Herein, we explored the molecular mechanism of PT by which it inhibited the cellular proliferation in colorectal cancer cells. We demonstrated for the first time that this platinum complex (PT) inhibited the colony forming ability of human colorectal cancer cells in a dose dependent manner. A similar polyamine complex of Pt(II) inhibited colony formation in breast cancer cells [46] and Pt (II) complex of 1, 10-phenanthroline exhibited antitumor effect in lung cancer [47]. Inhibition of cell proliferation is mainly mediated by apoptosis that plays an important role in fighting cancer. Therefore, for cancer therapeutic strategies apoptosis is a popular target. Induction of apoptosis by anticancer therapy is considered to be the most popular strategy to kill cancer cells [48]. In this study, we found that PT could trigger a significant increase in apoptosis in HT-29 and SW620 cells whereas induction of cell death in SW480 cell was found to be mediated by necrosis. ROS production plays an essential role in anticancer drug discovery. Previous evidence has suggested that some organometallic complex induced oxidative stress [49, 50]. In corroboration with these finding we found that PT induced ROS production and glutathione depletion in HT-29, SW480 and SW620 cells. Interestingly, the ROS production was much higher in HT-29 and SW620 cells than SW480 cells. Cancer cells are known to produce higher amount of ROS than normal cells, treating cancer cells with these ROS generating agents would insult these cells and leads to cell death [51]. The redox balance has been shown to play important role in cancer progression [52]. Higher ROS generation leads to alteration in mitochondrial membrane potential. In this study we found that PT treatment inhibited mitochondrial membrane potential in human colorectal cancer cells. Bcl2 family proteins which are grouped into anti-apoptotic protein (Bcl2, BclxL, Mcl1) and pro-apoptotic protein (Bax, Bak, Bid) play essential role in the regulation of early events of apoptosis [53]. The balance between anti-apoptotic and pro-apoptotic proteins determines the fate of cancer cells. The overexpression of Bcl2 and BclxL are known to block apoptosis and promote cancer cell proliferation, thus making them as attractive targets for cancer drug discovery. In this study we found that PT inhibited the expression of Bcl2, BclxL and XIAP in colorectal cancer cells. Therefore, inhibition of Bcl2 and BclxL makes cancer cells more vulnerable to apoptosis. Cyclins are known to play pivotal role in the regulation of cell cycle [31]. This platinum complex inhibited cyclin D1 expression and partially upregulated cyclin E1 expression at lower concentration of 5 μM while downregulated 10 μM. The significance of concentration dependent regulation of cyclin E1 by PT needs further exploration. Under stress pro-apoptotic Bax gets activated by oligomerization which leads to the release of cytochrome c from mitochondria into cytosol. The release of cytochrome c leads to the activation of caspase cascade and apoptosis [33, 53]. PT treated colorectal cancer cells were found to have increased amount of cytochrome c as compared to control cells. Cytochrome c release into cytosol leads to caspase-9 activation which in turn activates caspase-3. Activation of caspase family of proteins play essential role in the initiation and execution of apoptotic process [53]. Caspase-3 is the major rate limiting caspase resulting in the cleavage of PARP that leads to apoptotic cell death. PARP cleavage was detected in PT-treated colorectal cancer cells. We found that PT complex induced caspase-9, caspase-3 and caspase-7 activation in dose dependent manner. However, no caspase-8 activation was detected suggesting that PT induced intrinsic apoptotic pathway in colorectal cancer cells. Similar findings were obtained with ONS-donor ligand based Pt(II) complex which induced anticancer activity by PARP cleavage, Caspase-3/− 7 activation and autophagy [54]. Kowalski et al. reported that Oxidovanadium(IV) coordination complex induces cell cycle arrest and autophagy by ROS generation and triggering P53/21 pathway [55]. Our findings indicate that PT induces cell death by inhibition of anti-apoptotic proteins, increasing the cytochrome c release and PARP cleavage. We provided direct evidence that the production of ROS represented the first step of PT-induced apoptosis. N-acetylecysteine (NAC) is a ROS scavenger known to block ROS-mediated apoptosis. In fact, pre-treatment with NAC attenuated PT-induced cell death in human colorectal cancer cells.

Mitogen-activated protein kinases (MAPKs) belong to a highly conserved family of serine/threonine kinases. MAPK signaling plays a significant role in cancer cell proliferation, differentiation, motility, survival and apoptosis [17–19]. The family of MAPK is made up of JNK, p38 MAPK and ERK1/2 and known to regulate many of the cellular processes associated with growth, survival and cell death. We found that pre-incubation with p38 MAPK inhibitor (SB202190) significantly inhibited the PT-induced cell death in HT-29, SW480 and SW620 cells. AKT inhibitor inhibited PT-induced cell death significantly in HT-29 and SW480; whereas it has no significant effect in SW620 cells. Other inhibitors of MAPK family, like JNK and ERK had no effect on PT-induced apoptosis in colorectal cancer cells. This study thus indicates that PT-induced apoptosis is mediated by the activation of p38 MAPK signaling. AKT pathway plays a role in PT-induced cell death in HT-29 and SW480 but not in SW620 cells suggesting its involvement in a cell context dependent manner. In addition, we confirmed that PT induced the phosphorylation of p38 MAPK and inhibited the AKT phosphorylation in colorectal cancer cells. The phosphorylation of other MAPK like JNK and ERK was unperturbed in PT-treated cells. Silva et al. [56] showed that Ru(II)-thymine complex causes DNA damage and p38 activation in colorectal cancer similar to our finding. Consistent with these findings we found that PT-induced activation of p38 MAPK signaling leads to the phosphorylation of downstream substrate (HSP27-Ser82) in colorectal cancer cells. In addition, p38 MAPK inhibitor SB202190 blocked PT-induced cytochrome c confirming the role of p38 MAPK in PT-induced apoptosis. Furthermore, silencing of p38 by siRNA also inhibited PT-induced apoptosis in colorectal cancer cells supporting our findings from p38 MAPK inhibitor SB202190. PT may inhibit inflammatory pathway like NFkB and STAT3. NFkB and STAT3 pathway play essential role in inflammatory response and link chronic inflammation to colorectal cancer [57–59]. These pathways are known to regulate cell proliferation, apoptosis, invasion, metastasis and drug resistance. NFkB and STAT3 transcription factors regulate anti-apoptotic gene expression of Bcl2 and BclxL. Our finding indicates that PT inhibited Bcl2 and BclxL protein expression in various colorectal cancer cells indicating that PT may alter NFkB and STAT3 pathway. Zhu et al. have shown that cyclometalated Pt complex inhibited the cell viability of HepG2 cell without affecting normal cells by hijacking the NFkB pathway [60]. Others have reported the cytotoxic effectiveness of platinum complexes by inhibiting NFkB proteins-platinated DNA interaction [61].

With increasing incidence and high mortality associated with colorectal cancer specifically with advance disease stages, novel cancer therapeutics are needed urgently. Traditional chemotherapy against colorectal cancer includes oxaliplatin and other analogues of cisplatin. For decades, platinum complexes have been used as gold standard for anti-cancer therapy. However, treatment with platinum complexes resulting in higher side effect causing damage to healthy cells cannot be neglected. Many cancer cells exhibited inherent or acquired resistance to platinum-based drugs and prevent further clinical utilization. PT based on salicylaldimine ligand exhibited anticancer potential not only on adenocarcinoma colorectal cancer but also on metastatic colorectal cancer cells by generating ROS production and regulating MAPK pathway. Therefore, this platinum complex may be explored with improved cytotoxicity and minimal side effects in colorectal cancer patients.

## Conclusions

Our study for the first time elucidated the molecular mechanism of Pt(II) complex based on salen ligand-mediated inhibition of cell proliferation. PT could efficiently induce apoptosis in colorectal cancer cells. PT causes oxidative stress that triggers apoptosis mediated by p38 MAPK/AKT signaling independent of JNK and ERK. Our findings suggest that PT complex has the potential to be developed as an antitumor agent that may serve as an effective therapeutic addition to the current armamentarium in the management of colorectal cancers.

## Supplementary information

**Additional file 1: Supplementary Figure S1A-C.** Original western blots used in Fig. 5a, c, e. The cropping of the blot was clearly mentioned with red rectangle.

**Additional file 2: Supplementary Figure S2A-B.** Original western blots used in Fig. 6d-e. The cropping of the blot was clearly mentioned with red rectangle.

**Additional file 3: Supplementary Figure S3A-D.** Original western blots used in Fig. 8a-d. The cropping of the blot was clearly mentioned with red rectangle.

**Additional file 4: Supplementary Figure S4A-C.** Original western blots used in Fig. 9a-c. The cropping of the blot was clearly mentioned with red rectangle.

## Data Availability

All data generated or analyzed during this study are included in this paper.

## Abbreviations

ATCC: American Type Culture Collection
NAC: N-Acetylcysteine
CRC: Colorectal Cancer
DC-FDA: 2′,7′-dicholorodihydrofluorescein diacetate
PI: Propidium Iodide
ROS: Reactive Oxygen Species
MAPK: Mitogen activated protein kinases
