# Correction to: A novel coordination complex of platinum (PT) induces cell death in colorectal cancer by altering redox balance and modulating MAPK pathway

**DOI:** 10.1186/s12885-020-07245-x

**Published:** 2020-09-01

**Authors:** Khayal Al-Khayal, Mansoor-Ali Vaali-Mohammed, Mohammed Elwatidy, Thamer Bin Traiki, Omar Al-Obeed, Mohammad Azam, Zahid Khan, Maha Abdulla, Rehan Ahmad

**Affiliations:** 1 Colorectal Research Chair, Department of Surgery, King Saud University College of Medicine, PO Box 7805 (37), 11472 Riyadh, Saudi Arabia; 2 College of Medicine Research Center, King Saud University College of Medicine, Riyadh, 11472 Saudi Arabia; 3 Department of Chemistry, College of Science, King Saud University, Riyadh, 11451 Saudi Arabia; 4 Genome Research Chair, Department of Biochemistry, College of Science, King Saud University, Riyadh, Saudi Arabia

**Correction to: BMC Cancer 20, 685 (2020)**

**https://doi.org/10.1186/s12885-020-07165-w**

Following publication of the original article [1], the authors identified a typesetting error in Fig. 2, whereby the full figure was not published. The complete Fig. 2 is published in this correction article and the original article [1] has been corrected.

**Fig. 2:**
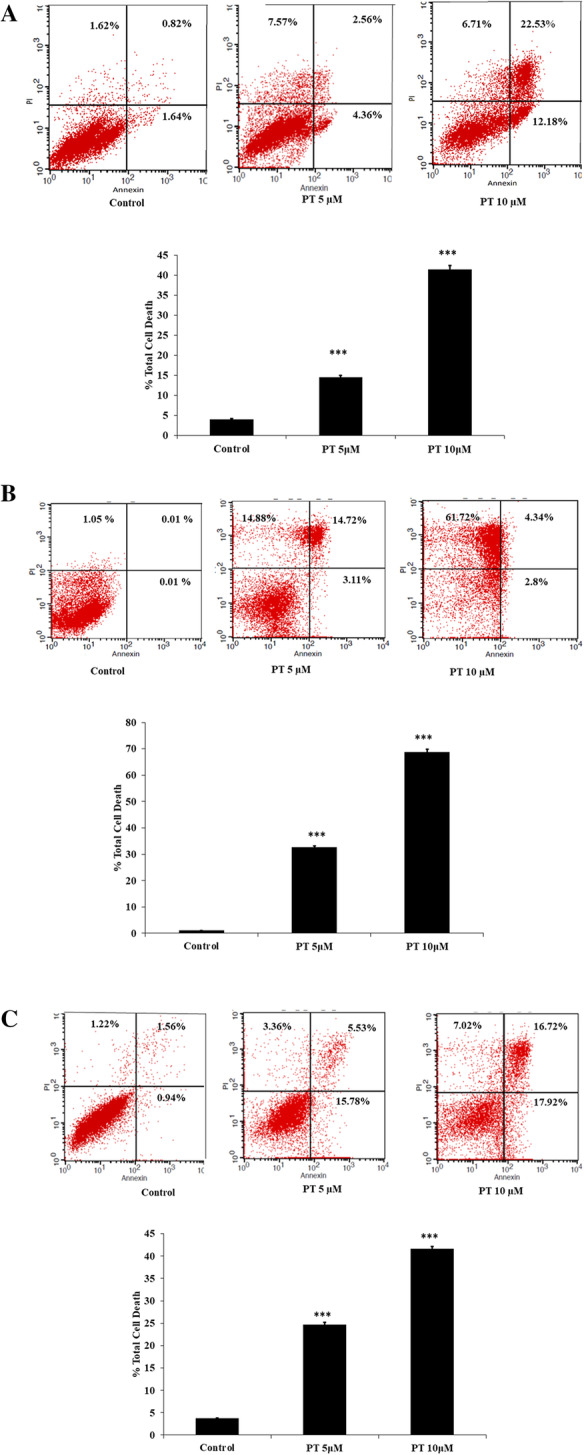

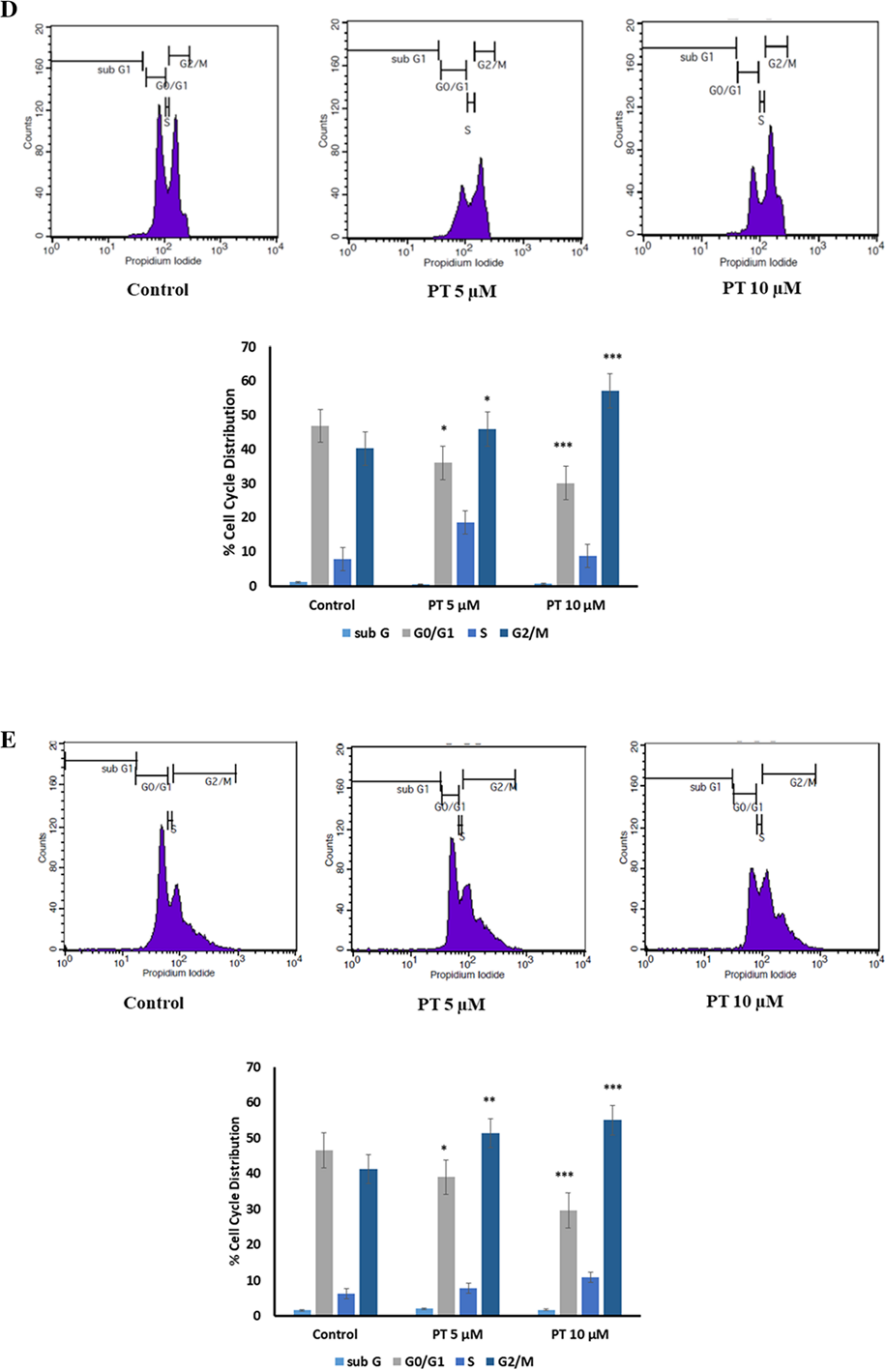
PT induces apoptosis and cell cycle arrest. **a** HT-29 **b** SW480 **c** SW620 cells were treated with 5 and 10 μM of PT for 24 h. Total cell death including apoptosis and necrosis was analyzed by Annexin V/PI staining using flow cytometry. **d** HT-29 and **e** SW620 cell cycle distribution was measured by PI staining using flow cytometry and the percentage of cell population was determined in the G0/G1, S and G2/M phases. Results shown are representative of three independent experiment (*n* = 3). *p < 0.05, **p < 0.01, ***p < 0.001 vs control
