# Supplementary figures and images for: A novel coordination complex of platinum (PT) induces cell death in colorectal cancer by altering redox balance and modulating MAPK pathway
Source: BMC Cancer. 2020 Jul 23;20:685. doi: 10.1186/s12885-020-07165-w (PMC7376665; doi:10.1186/s12885-020-07165-w)

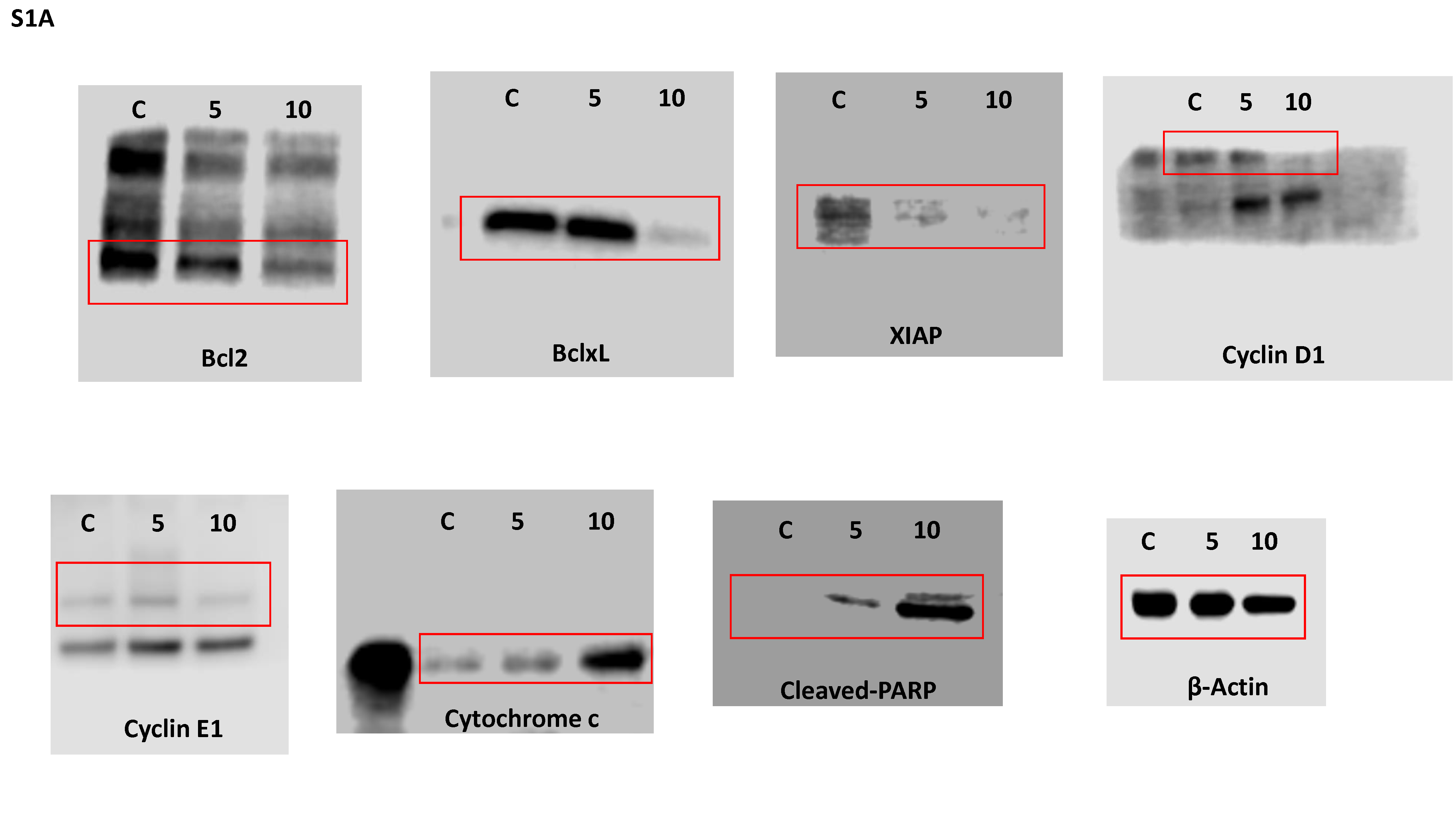

Supplement: Supplementary file 1 — Additional file 1: Supplementary Figure S1A-C. Original western blots used in Fig. 5a, c, e. The cropping of the blot was clearly mentioned with red rectangle. [file 12885_2020_7165_MOESM1_ESM.tiff]

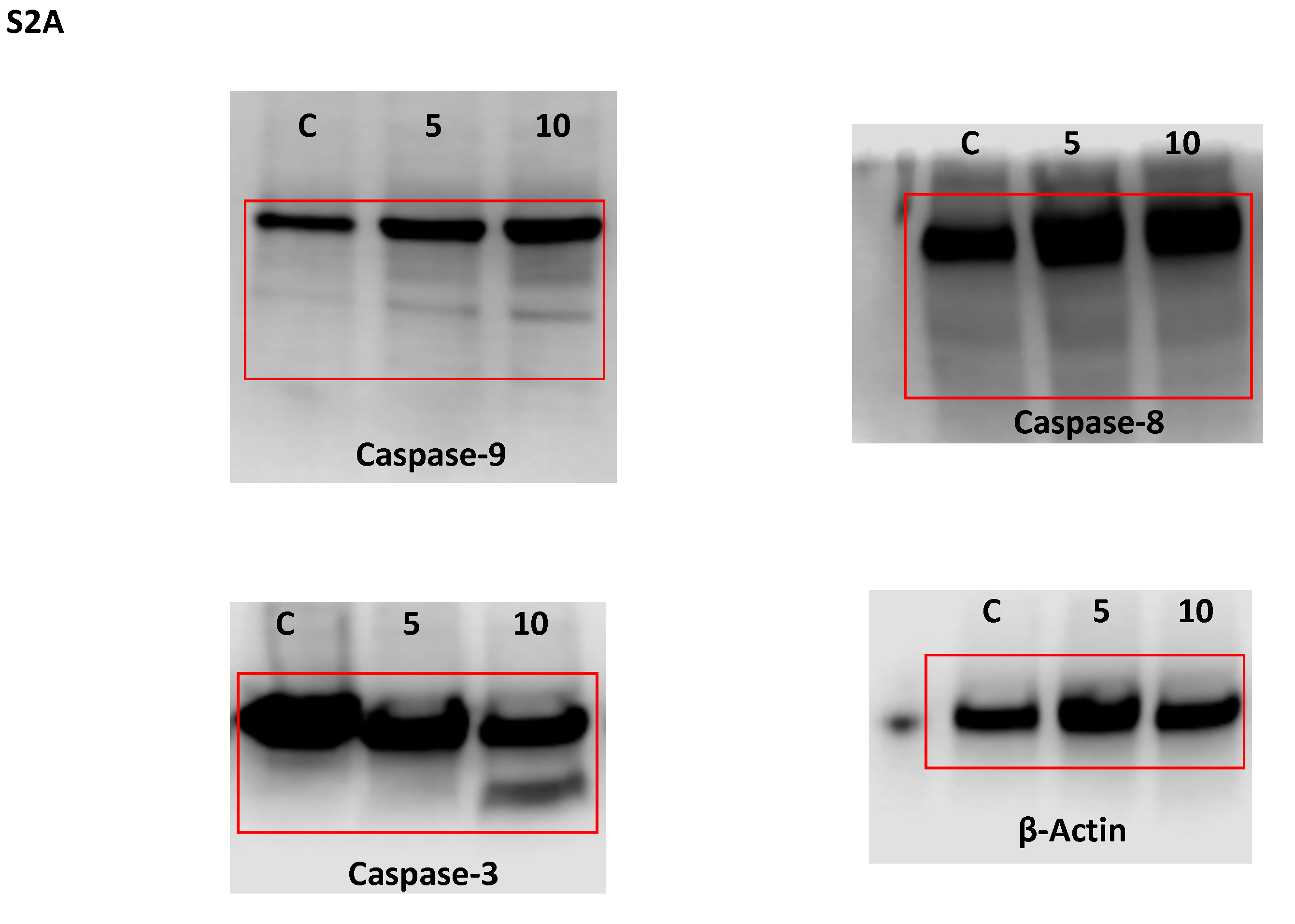

Supplement: Supplementary file 2 — Additional file 2: Supplementary Figure S2A-B. Original western blots used in Fig. 6d-e. The cropping of the blot was clearly mentioned with red rectangle. [file 12885_2020_7165_MOESM2_ESM.tiff]

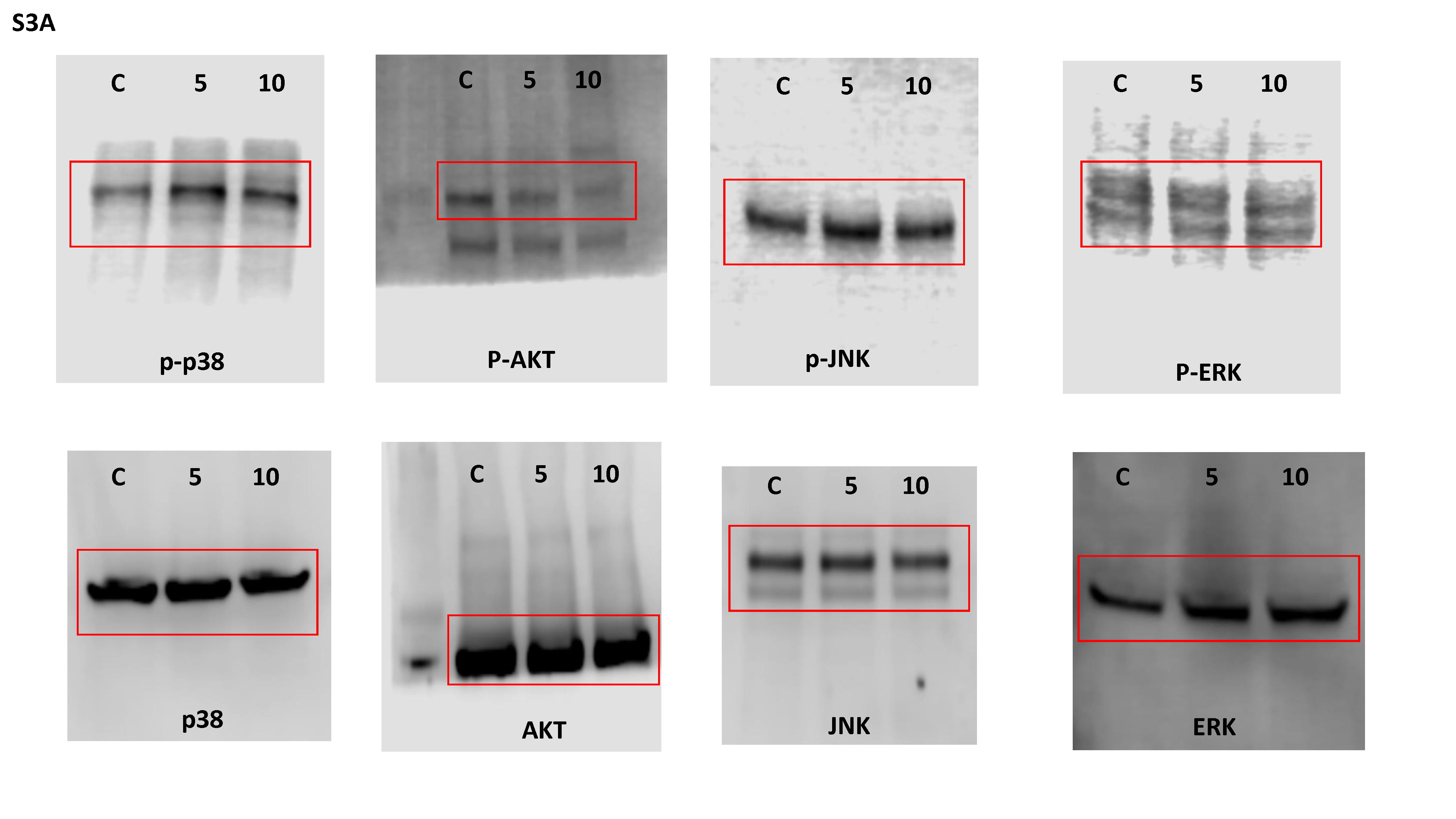

Supplement: Supplementary file 3 — Additional file 3: Supplementary Figure S3A-D. Original western blots used in Fig. 8a-d. The cropping of the blot was clearly mentioned with red rectangle. [file 12885_2020_7165_MOESM3_ESM.tiff]

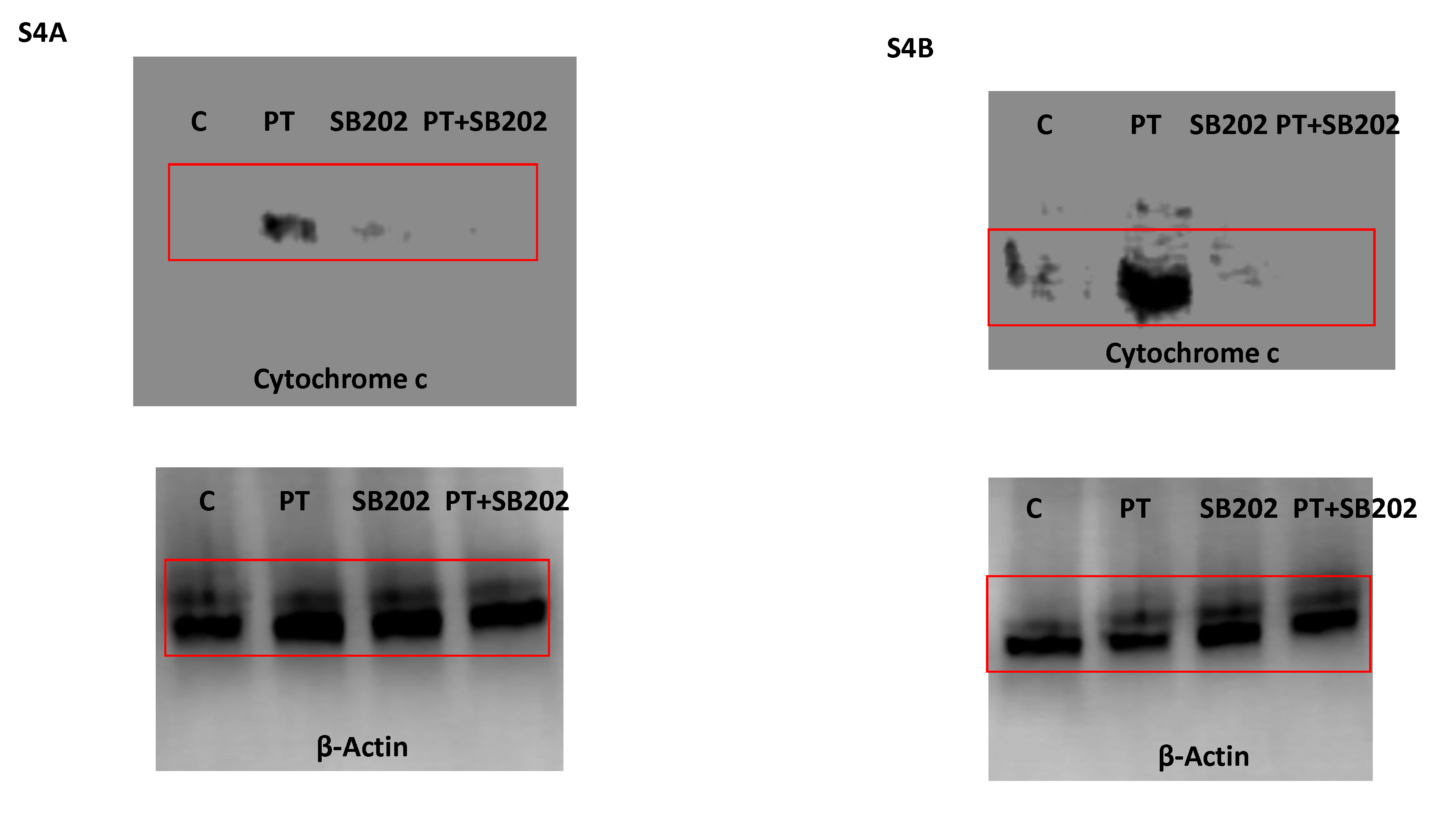

Supplement: Supplementary file 4 — Additional file 4: Supplementary Figure S4A-C. Original western blots used in Fig. 9a-c. The cropping of the blot was clearly mentioned with red rectangle. [file 12885_2020_7165_MOESM4_ESM.tiff]
